# Hybrid Kerr-electro-optic frequency combs on thin-film lithium niobate

**DOI:** 10.1038/s41377-025-01906-x

**Published:** 2025-08-12

**Authors:** Yunxiang Song, Yaowen Hu, Marko Lončar, Kiyoul Yang

**Affiliations:** 1 John A. Paulson School of Engineering and Applied Sciences, Harvard University, Cambridge, MA USA; 2 Quantum Science and Engineering, Harvard University, Cambridge, MA USA

**Keywords:** Frequency combs, Integrated optics, Photonic devices

## Abstract

Optical frequency combs are indispensable links between the optical and microwave domains. Chip-scale integration promises compact, scalable, and power-efficient frequency comb sources, enabled by the resonantly-enhanced Kerr effect or the electro-optic effect. While combs utilizing the former can reach octave-spanning bandwidths, and combs based on the latter can feature microwave-rate spacings, achieving both features at the same time has been challenging. Here, we simultaneously leverage the strong Kerr and electro-optic effects on thin-film lithium niobate, where dissipative Kerr soliton generation is followed by electro-optic phase modulation, to realize an integrated frequency comb reference with 2,589 lines spaced by 29.308 GHz and spanning 75.9 THz (588 nm). Further, we demonstrate electronic stabilization and control of the comb spacing, naturally facilitated by this approach. The broadband, microwave-rate frequency comb in our work overcomes the spacing-span tradeoff that exists in nonlinear integrated frequency comb sources, paving the way towards chip-scale solutions for next-generation laser spectroscopy, microwave and millimeter wave synthesis, as well as optical communications.

## Abstract (web summary)

We demonstrate a hybrid approach to generating chip-scale microcombs leveraging Kerr and electro-optic nonlinearities of thin-film lithium niobate, reaching 2589 comb lines spaced by 29.308 GHz and spanning 75.9 THz.

## Graphical abstract

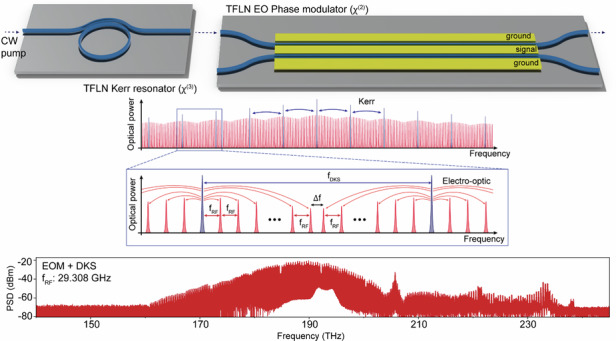

## Introduction

The generation and stabilization of optical frequency combs have had a significant impact on scientific and industrial advancements. Optical atomic clocks^1^, ultrafast lasers^2,3^, and precision spectroscopy^4,5^ have been developed based on this foundational technology. Currently, the demand for comb-driven systems has intensified, accompanied by stringent constraints on their scalability and operational budgets. Chip-scale integration, based on low-loss and highly nonlinear nanophotonic devices, has shown potential to reduce power consumption and system volume by orders of magnitude^6^. Recent demonstrations, which critically relied on chip-scale frequency comb sources^7–9^, showcased several advanced capabilities including frequency division of optical carriers down to microwaves^10–14^, optical frequency synthesis^15^, frequency multiplexed computing^16–18^ and communications^19–21^, as well as dual-comb spectroscopy^22^. Continuous development of the integrated comb sources will result in major enhancements to such demonstrations, which can substantially benefit from microwave-rate comb spacings while simultaneously featuring broad comb spans. In particular, microwave-rate spacings provide densely spaced frequency channels for data transmission and processing. Additionally, such spacings can directly link rapidly oscillating optical frequencies to electronically detectable microwaves. At the same time, broad comb spans enable spectroscopy, as well as optical frequency metrology and synthesis, over large spectral ranges. However, current integrated frequency comb sources, such as dissipative Kerr solitons (DKSs) and electro-optic (EO) frequency combs^8,23^, are limited by a spacing-span tradeoff^8,9^. While the DKS can feature up to octave-spanning bandwidths, the tradeoff between nonlinear enhancement and comb spacing dictates that octave-spanning combs have comb lines separated by hundreds of GHz or more^15,24–27^. On the other hand, EO frequency combs have spacings directly set by the microwave-rate modulation frequencies, but achievable comb spans are limited by the maximally attainable EO interaction strength, optical quality factor, and dispersion properties^28–31^.

To overcome these limitations, prior works have utilized a combination of material nonlinearities. Two leading approaches involve the EO pulse-pumping of nonlinear microresonators^32,33^ and the cascading of Kerr combs with EO modulation^34–36^. In the first approach, discrete EO amplitude and phase modulators, paired with dispersion compensating fiber, produce high peak power pulses. These pulses are then used to initiate broadband combs featuring microwave-separated lines, assisted by a nonlinear microresonator. The second approach involves sending Kerr combs through bulk EO modulators, the latter dividing large Kerr comb spacings down to the microwave level. While both approaches have successfully achieved microwave-rate comb spacings and large comb spans, full system integration of these approaches lacks chip-scale elements for high peak power pulse generation, low-loss pulse compression, and efficient EO modulation. Beyond hybrid approaches, integrated frequency comb generation leveraging the strong Kerr and EO nonlinearities of thin-film lithium niobate (TFLN) was explored, leading to discoveries of independent DKS and resonant EO comb^37^, EO-tunable DKS^38^, and EO-Raman comb^31^, but all still face major bandwidth limitations. Therefore, the generation of comb spectra with simultaneously large span and low spacing remains a critical hurdle for the field of integrated frequency combs.

Here, we demonstrate a hybrid Kerr-EO frequency comb with microwave-rate spacing (29.308 GHz) and broad bandwidth (75.9 THz). In our approach, the resonance-enhanced Kerr effect produces a mode-locked, broadband, and near THz-rate DKS frequency comb. Subsequent non-resonant EO comb generation is used to coherently densify the DKS spacing down to microwave frequencies across its original bandwidth. We separately establish Z-cut TFLN as a promising DKS platform owing to the photorefraction-induced operational simplicity and robustness, as well as octave-spanning capability, and X-cut TFLN as an ultra-efficient EO platform because of the large EO coefficient and confined microwave-optical interaction. Combining these unique properties, hybrid Kerr-EO combs implemented on TFLN feature many advantages, including compact form factor, reduced power requirements, exceptional free-running uptime, and strong nonlinear optical efficiency, all facilitated by photonic integration. Thus, our result offers a clear path forward towards broadband, microwave-rate frequency combs on a chip.

## Results

### Device design and experimental approach

**Fig. 1:**
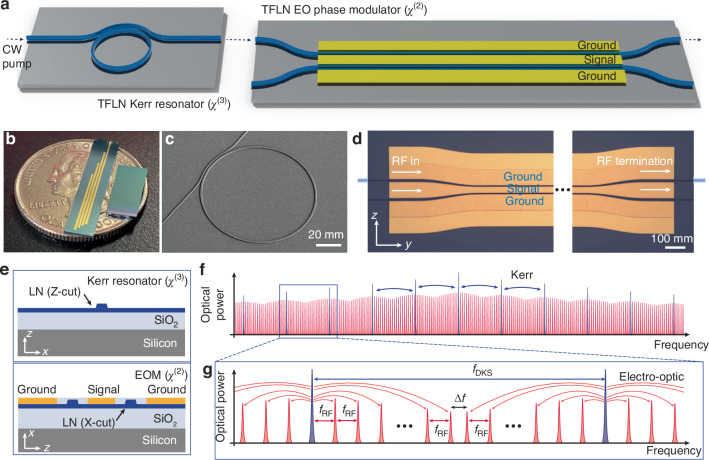
**Concept of hybrid Kerr-electro-optic frequency comb.** **a** 3-D illustration of the hybrid Kerr-EO frequency comb, consisting of a DKS microresonator chip and an EO phase modulator chip. A continuous-wave (CW) optical frequency initiates a near THz-rate DKS frequency comb, which then undergoes EO phase modulation to form a microwave-rate hybrid comb after passing through the modulator. **b** Photograph showing TFLN photonic chips with EO phase modulator array (left) and Kerr resonators (right). **c** Scanning electron microscope image of the Kerr resonator. **d** Optical microscope image of two parallel EO phase modulators. In this work, we use only one phase modulator, where one optical waveguide passes through the ground-signal gap of the coplanar line. **e** Cross-sectional schematic of the Kerr resonator (top) and the EO phase modulator (bottom), with crystal axes labeled to indicate Z- and X-cut, respectively. **f** Schematic of the hybrid Kerr-EO frequency comb generation process. A DKS frequency comb (blue) is used as a source, where each comb line generates EO sidebands (red) around it at multiples of the modulation frequency. The final output of the hybrid comb generator consists of both blue and red lines. **g** Schematic of the variables defined in our work. ${f}_{{DKS}}$: DKS spacing, ${f}_{{RF}}$: hybrid Kerr-EO comb spacing and EO modulation frequency, $\Delta f$: difference frequency defined by ${f}_{{DKS}}=N\cdot {f}_{{RF}}+\Delta f$

Our hybrid Kerr-EO approach for frequency comb generation on TFLN cascades a dispersion-engineered microresonator and a high-speed, non-resonant EO comb based on phase modulation. The microresonator outputs a DKS with a comb spacing in the range of hundreds of GHz. Each soliton line acts as a source for phase modulation, resulting in EO sidebands generated around each DKS comb line. As such, the original DKS comb spacing is divided down to a microwave-rate spacing set by the EO modulation frequency. The integrated components required are schematically shown in Fig. 1a. Utilizing this approach, we developed anomalous dispersion Kerr microresonators and EO phase modulators on Z-cut and X-cut TFLN chips, respectively. Images and schematics of the fabricated devices and cross sections are shown in Fig. 1b–e. The working principle of the hybrid Kerr-EO approach and the hybrid comb output are illustrated in Fig. 1f, g. We note that the TFLN photonic platform is suitable for implementing the hybrid Kerr-EO approach, due to its large Kerr and EO nonlinearities.

### DKS on Z-cut TFLN

**Fig. 2:**
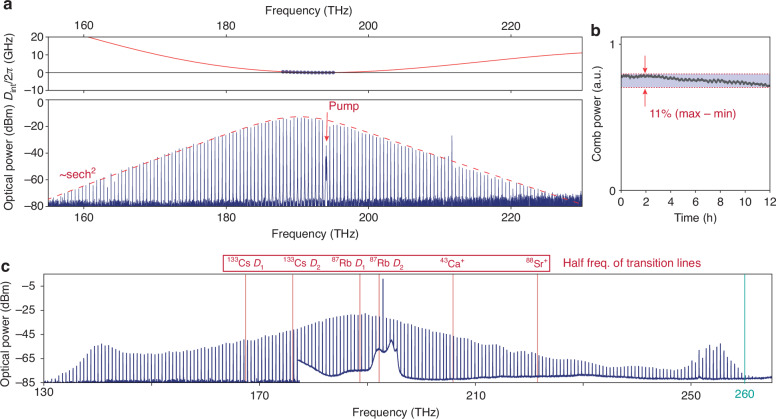
**Dissipative Kerr solitons on Z-cut thin-film lithium niobate.** **a** Simulated (red curve) and measured (blue dots) integrated dispersion of an anomalous dispersion microresonator (top) and the associated single DKS spectrum (bottom), showcasing a prototypical sech^2^ envelope with a spacing between comb lines (${f}_{{DKS}}$) of ~504 GHz. The pump frequency is ~194 THz and is removed using a fiber-Bragg-grating notch filter after the chip. The amplified spontaneous emission noise associated with pump amplification is filtered out using a tunable bandpass filter before the chip. **b** Free-running stability of the single DKS state in **a** once manually initiated. The DKS comb power remains steady over 12 hours without any active control, and its smooth decrease (over 11% of its maximum) is dominated by the slowly drifting lensed-fiber coupling on and off the chip. The comb power is normalized to the photodetector dynamic range (Supplementary information Fig. 2). **c** Octave spanning single DKS spectrum covering 131.3–263.2 THz end-to-end with ${f}_{{DKS}} \sim 660$ GHz. Half-frequencies of six atomic transition lines are overlaid (red lines), indicating sufficient spectral coverage over stable atomic transitions for comb stabilization

Z-cut TFLN supports low-loss microresonators^39,40^ hosting broadband DKS states that can be initiated manually^41^. Remarkably, the DKSs on Z-cut TFLN also exhibit excellent free-running stability^42^. In contrast to the thermo-optic self-stability of microresonators^43^ when driven by a blue-detuned pump, common to nearly all other photonic platforms (including X-cut TFLN^44^), microresonator resonances on this platform stabilize against fluctuations in the pump frequency when the pump is red-detuned. This detuning regime coincides with the region of DKS existence, thus simplifying the DKS generation process compared to other platforms, where thermo-optic instability of red-detuned pumps hinders adiabatic tuning into DKS states^45^. In Fig. 2a, we show a prototypical single DKS state operating in the fundamental transverse-electric-like mode. A strong quadratic term dominates the total microresonator dispersion, giving rise to a purely sech^2^ spectrum under a moderate on-chip pump power of 189 mW. Once this DKS state is manually initiated, it is self-stable as evidenced in Fig. 2b by the finite comb power measured continuously over 12 hours. We note that no temperature control of the chip, nor feedback acting on the microresonator or the pump laser, is performed during this time.

To further demonstrate the potential of DKSs on Z-cut TFLN, we demonstrate an octave-spanning single DKS frequency comb extending from 131.3 THz to 263.8 THz, featuring a spacing of 660 GHz and manually initiated under 372 mW of on-chip pump power, as shown in Fig. 2c. This is enabled by dispersion engineering via precisely tuning the radius, waveguide width, and waveguide height (etch depth) of the TFLN microresonator. We also demonstrate the well-controlled dispersion engineering of near-octave DKSs broadened by dual dispersive waves (Materials and methods, Supplementary information Fig. S1), which can be manually initiated under moderate on-chip pump powers as well. Such DKSs serve as ideal sources for practical hybrid Kerr-EO frequency combs.

### EO comb on X-cut TFLN

**Fig. 3:**
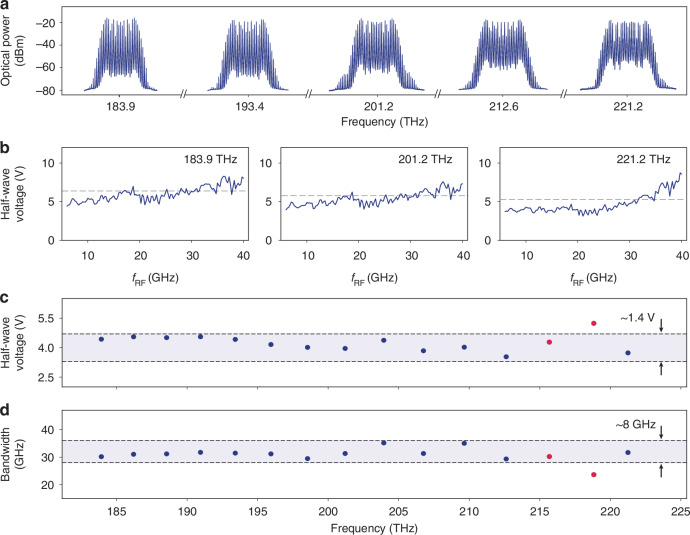
**Integrated electro-optic phase modulator characterization on X-cut thin-film lithium niobate.** Integrated electro-optic phase modulator characterization on X-cut thin-film lithium niobate. **a** EO frequency combs generated by a 2 cm-long, integrated EO phase modulator at pump frequencies of 183.9, 193.4, 201.2, 212.6, and 221.2 THz (equivalent wavelengths of 1630, 1550, 1490, 1410, and 1355 nm). The modulation frequency is 29.158 GHz, and the electrical power is estimated to be 4.47 W (36.5 dBm). **b** Half-wave voltage (${V}_{\pi }$) at different modulation frequencies (blue curve) and the $\sqrt{2}$-scaled ${V}_{\pi }$ referenced to the 6 GHz ${V}_{\pi }$ (gray dashed line, corresponding modulation frequency is the 3 dB EO bandwidth), at three select optical frequencies. **c** Optical frequency dependence of the 6 GHz ${V}_{\pi }$ and **d** the 3 dB EO bandwidth. The 6 GHz ${V}_{\pi }$ and 3 dB EO bandwidth variation (blue shaded region enclosed by black dashed lines) over ~30 THz optical bandwidth is about 1.4 V and 8 GHz, respectively. Points near 215 and 219 THz (red dots) are affected by mode-crossings and have relatively higher 6 GHz ${V}_{\pi }$ and lower 3 dB EO bandwidth

Next, we characterize the second piece of the hybrid comb generation approach. An X-cut TFLN EO phase modulator is operated at multiple wavelengths sampled within the source DKS bandwidth to generate non-resonant EO combs. A 2 cm-long coplanar line is used to induce a large optical phase shift and reduce the ${V}_{\pi }$, while simultaneously exhibiting a large microwave bandwidth^46,47^. Single-tone microwaves at 29.158 GHz are continuously delivered to the coplanar line for performance characterization. It is important to note that the EO phase modulator here operates over a broad, uninterrupted microwave band and a continuous optical frequency span, compared to cavity-based EO comb generators^37^. Figure 3 showcases the broadband compatibility of the EO phase modulator for both microwave and optical frequencies. Fifteen CW optical frequencies in the telecommunications L, C, S, and E bands are separately coupled onto the chip, and EO sidebands are generated around each frequency. Five representative examples are shown in Fig. 3a. At all optical frequencies, the microwave power applied is estimated to be 4.47 W (36.5 dBm), showcasing the efficiency and power-handling ability of our modulator. We determine the EO phase modulator bandwidth at each wavelength by measuring the modulation-frequency-dependent ${V}_{\pi }$ (see Materials and Methods for details), results shown in Fig. 3b–d. While the ${V}_{\pi }$ and EO bandwidth show slight dependencies on the optical frequency, likely due to varying optical-microwave spatial mode overlaps and the optical frequency itself^48^, they are sufficiently uniform considering the total bandwidth of our DKS combs and to achieve the required number of EO sidebands for complete EO division of their spacings down to microwave frequencies.

### Hybrid Kerr-EO integrated frequency comb

**Fig. 4:**
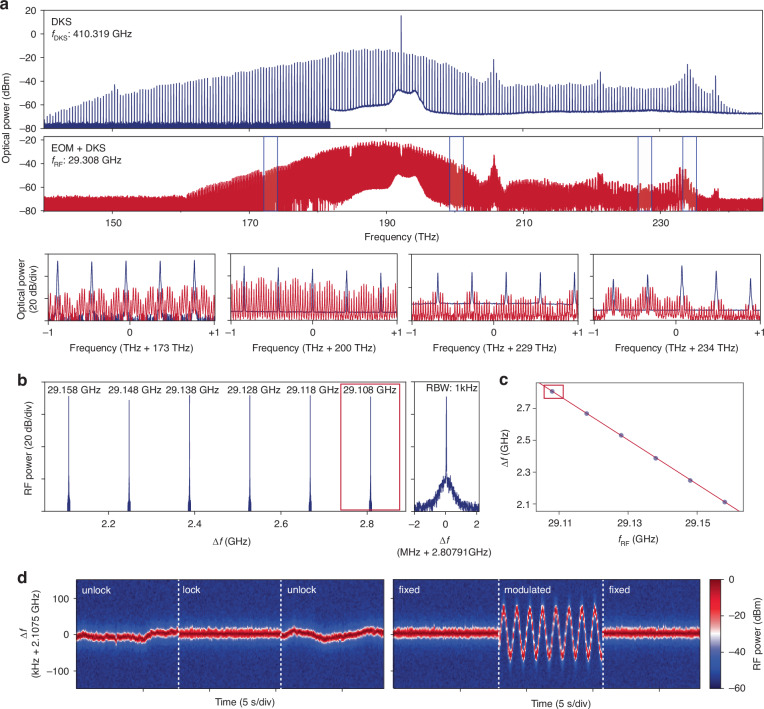
**Hybrid Kerr-electro-optic frequency comb on thin-film lithium niobate.** **a** Single DKS spectrum (blue, top) with a spacing ${f}_{{DKS}}$ of 410.319 GHz between comb lines and electro-optically modulated single DKS spectrum (red, bottom) with a 14-times divided spacing of 29.308 GHz. The latter spectrum is zoomed-in over 2 THz windows centered ~173, 200, 229, and 234 THz, with original DKS comb line positions overlaid. **b** Nonzero difference frequencies $\Delta f$ detected as a beatnote, as the EO modulation frequency is stepped from 29.108 GHz up to 29.158 GHz in intervals of 0.01 GHz. When the modulation frequency is 29.108 GHz, a zoom-in of the beatnote over a 4 MHz window centered around 2.80791 GHz shows a narrow linewidth with 1 kHz resolution bandwidth. **c** Linear fit of the difference frequencies $\Delta f$ to the modulation frequencies (${f}_{{RF}}$) in **b**. The absolute value of the slope is $N=14$, the number of EO sidebands required to completely divide the single DKS spacing. **d** Spectrogram of $\Delta f$ when ${f}_{{RF}}$ is fixed at 29.158 GHz. Left panel shows the progression of $\Delta f$ when it is unlocked, phase locked to a microwave reference oscillator, and unlocked again. Right panel shows the progression of $\Delta f$ when it is phase locked to a microwave reference oscillator that is fixed frequency, sinusoidally modulated in frequency, and fixed frequency again

Combining self-starting and stable DKSs with efficient EO phase modulation, we realize a chip-integrated hybrid Kerr-EO comb. By sending a 410.319 GHz-spaced DKS into the EO phase modulator, we obtain a hybrid Kerr-EO comb spanning 75.9 THz end-to-end with a spacing of 29.308 GHz (Fig. 4a). The relationship between the comb spacings is given by ${f}_{{DKS}}=N\cdot {f}_{{RF}}+\Delta f$, where ${f}_{{DKS}}$ and ${f}_{{RF}}$ are the DKS and hybrid Kerr-EO comb spacings, respectively, *N* is the number of EO sidebands to fully divide one DKS spacing, and $\Delta f$ is the difference frequency provided ${f}_{{RF}}$ is not a perfect subharmonic of ${f}_{{DKS}}$. These quantities are also labeled in Fig. 1g. Note that *N* is the largest integer for which $\left|\Delta f\right|\, < \, {f}_{{RF}}$ holds. When the EO sidebands perfectly divide the DKS spacing, ${f}_{{DKS}}/{f}_{{RF}}=N$ and $\Delta f=0$. The DKS spectrum is filled in by microwave-separated EO comb lines, as shown in the zoom-in windows of Fig. 4a. Power variation in the comb lines within each window is due to the EO sideband envelope determined by the local modulation depth, and variation between windows comes from the optical-frequency-dependent ${V}_{\pi }$. The reduction in spectral span of the hybrid Kerr-EO comb compared to the original DKS is due to facet losses from the two chips, fiber link loss between the chips, and optical power redistribution into EO sidebands below the detection sensitivity. The optical pump power for DKS generation is 125 mW (21 dBm), and the electrical power to drive the EO phase modulator is 2.51 W (34.5 dBm), both on-chip. The full connectivity displayed by the hybrid comb offers densely positioned optical frequency references over its entire bandwidth. The free-running uptime of this broadband reference is solely determined by that of the source DKS, which we have previously shown can be more than 12 hours even in an uncontrolled environment. We note that while the EO modulation densifies DKS spacings in the frequency domain, it does not alter the time-domain intensity of the DKS pulse train.

Next, we set $\Delta f$ to be a nonzero value of around 2.107 GHz to elucidate the spacing relationship. As the microwave drive frequency (which sets ${f}_{{RF}}$) is linearly decreased in steps of 0.01 GHz, the $\Delta f$ linearly increases, as shown in Fig. 4b, c. Fitting ${f}_{{RF}}$ against $\Delta f$, the slope gives precisely $N=14$, in support of the spacing relationship. Confirming this relationship, the single integrated EO phase modulator thus demonstrated a direct measurement of the near THz spacing. Importantly, this THz metrology enabled by efficient EO down-conversion transduces the ${f}_{{DKS}}$ into a GHz difference frequency $\Delta f$ compatible with detection using fast electronics. Further, the detected $\Delta f$ may then be compared and phase locked to a stable microwave reference by feeding back onto the DKS pump frequency, which in turn stabilizes the original DKS spacing. In Fig. 4d, we phase lock the $\Delta f$ ~2.107 GHz to 32 times a microwave reference oscillator set at a frequency of 65.865 MHz. When unlocked, the $\Delta f$ fluctuates according to the fluctuation in ${f}_{{DKS}}$. When the lock is engaged, $\Delta f$ is pinned to a center frequency of 2.107 GHz with a linewidth of about 30 kHz. Sinusoidally modulating the reference oscillator frequency during the phase lock, the $\Delta f$ follows the reference modulation and so does ${f}_{{DKS}}$, indicating the detection-based, integrated EO control over the near THz DKS spacing. Such a method for DKS spacing control stands in contrast to EO-based parametric seeding techniques, which may only control microwave-rate, span-limited Kerr combs^38,49^ or, in principle, seed THz combs with very poor duty cycles. Thus, our hybrid Kerr-EO approach on TFLN not only generates broadband, microwave-rate combs, but also opens a route towards on-chip methods for the detection, stabilization, and control over large DKS spacings in the THz regime.

## Discussion

In summary, we presented a hybrid Kerr-EO approach for integrated frequency comb generation and demonstrated TFLN as a material platform that realizes this approach completely. Our work is enabled by the outstanding second and third-order nonlinear properties of TFLN, hosting uniquely self-starting and free-running DKSs with near THz spacings, while supporting EO phase modulation featuring broad optical and microwave bandwidth as well as high efficiency. We note that these characteristics are not provided by one crystal cut alone. Currently, the comb line power difference between the DKS source as extracted and the hybrid Kerr-EO comb is attributed to EO power redistribution into the sidebands (about 10 dB carrier suppression ~1550 nm), the off-chip coupling loss from the DKS chip (~6 dB), and the insertion loss of the EO modulator chip (~8 dB, both chip facets and propagation loss included). While the former is fundamental to the hybrid Kerr-EO approach, it is not a source of loss but rather a near-unity efficiency frequency conversion process, rendering phase modulation ideal compared to other sideband generation methods. The latter two losses, totaling 14 dB, can be reduced substantially so that the hybrid Kerr-EO comb bandwidth may approach the original DKS bandwidth. For example, integration between Z-cut and X-cut TFLN provides unique advantages compared to combining different materials^50^. In addition to advantages offered by hybrid integration (e.g., via photonic wirebonding, recently demonstrated on the TFLN platform^51^), novel integration of different TFLN crystal cuts into a single chip^52^ is feasible due to their similar waveguide cross-sections for DKS and EO applications, as well as their compatibility with the same material processing workflow (etching in particular), similar thermal properties, and so on. In our characterization setup, the excess fiber length and fiber bending between the chips led to ~12 dB additional attenuation in the <150 THz range due to silica absorption, a source of loss which is not fundamental to the hybrid Kerr-electro-optic approach and may be eliminated by advanced integration methods as previously mentioned. The monolithic integration of DKS and EO functionalities on either X-cut TFLN or Z*-*cut TFLN can eliminate interconnect losses in principle, but it requires substantial further exploration. Among many challenges, the in-plane birefringence can limit achievable DKS bandwidths on X-cut TFLN, and the largest EO coefficient is utilized by the fundamental transverse-magnetic-like mode (TM mode) on Z-cut TFLN. To this end, the X-cut TFLN platform is more promising, since reducing the initial film thickness of X*-*cut TFLN readily alleviates the birefringence-induced spectral limit, while efficient Z-cut TFLN EO modulators operating on the TM mode are not common due to the vertical gating requirement. Further, the electrical power requirement of driving the EO phase modulator may be reduced down to hundreds of milliwatts, employing advanced modulator designs but potentially trading off operational flexibility^53,54^. Finally, the hybrid Kerr-EO architecture may also synergize DKSs with advanced on-chip EO conversion devices such as high-efficiency resonant EO frequency combs^31^ to further reduce the electrical power requirement or increase the comb line density.

Our integrated comb source, serving as a broadband, microwave-rate frequency reference spanning 75.9 THz around the telecommunications band, is suitable for the measurement, control, and mutual stabilization of optical frequencies. Such frequencies may take the form of independent chip-scale lasers^55–60^ for gap-free and frequency-accurate laser spectroscopy^61–63^. Full stabilization of the hybrid Kerr-EO reference, such that the carrier-envelope offset frequency and comb spacing are simultaneously locked, can be achieved through a combination of Kerr-induced synchronization^36^, electronic feedback locking of a comb line to a stable atomic transition^64–66^, and interferometric self-referencing^15,67^ in conjunction with on-chip frequency doubling^68^, once the hybrid Kerr-EO comb surpasses an octave-span. The latter case would require increased pump-to-comb conversion efficiency for the DKS, or octave-separated dispersive wave emission, while also benefiting from heterogeneous and hybrid integration efforts applied to the DKS and EO chips. Importantly, the hybrid Kerr-EO comb’s microwave-rate spacing should ease the challenge of detecting and stabilizing a possibly near-THz carrier-envelope offset frequency. Indeed, the locking and modulation of a non-zero difference frequency $\Delta f$ in the hybrid Kerr-EO comb already enables direct stabilization and control of the large DKS spacing, which itself could already form the basis for THz-generation related applications such as radar and wireless communications^69,70^. Further, the hybrid Kerr-EO configuration may extend conventional optical^10–14^ and EO^71^ frequency division schemes towards integrated synthesizers of ultra-stable and tunable microwaves that promise orders of magnitude improvement in division factor. Therefore, the hybrid Kerr-EO frequency comb provides a foundation for next-generation integrated photonic devices, with the potential to play a key role in system-level applications where the capabilities of optical frequency combs are essential.

## Materials and methods

### Device fabrication and parameters

DKS devices are fabricated on a commercial Z-cut thin-film lithium niobate (Z-TFLN) on-insulator wafer (NANOLN). The initial film thickness is 600 nm of Z-TFLN on top of 2 μm thermal oxide and 0.525 mm silicon. Nanophotonic waveguides are patterned on hydrogen silsesquioxane (HSQ) resist using electron-beam lithography (EBL). The chip with patterned resist undergoes two iterations of Ar^+^-based reactive ion etching and wet etching in 75-degree SC-1 solution until an etch depth of ~345 nm is reached, leaving a slab thickness of about 255 nm. This iterative etching process results in a waveguide sidewall angle of about 72 degrees, owing to wet etchant removal of redeposition buildup during the dry-etching process. Finally, the HSQ resist is stripped using dilute hydrogen fluoride (HF), and the devices are annealed for two hours in a 520-degree oxygen-rich environment. Routing waveguides for coupling light on and off the chip are exposed through manual cleaving, resulting in coupling losses of about 6 dB per facet.

Electro-optic (EO) phase modulator devices are fabricated on a commercial X-cut thin-film lithium niobate (X-TFLN) on-insulator wafer (NANOLN). The initial film thickness is 600 nm of X-TFLN on top of 2 μm thermal oxide and 0.525 mm silicon. The fabrication process is identical to that for DKS devices up to etching of the waveguides (320 nm etch depth and 280 nm slab). Plasma-enhanced chemical vapor deposition (PECVD) is used to deposit a silica top cladding layer. Bilayer taper edge couplers are fabricated by first opening photolithography-defined rectangular windows in the top cladding using HF wet etching. Subsequently, the edge coupler tips are defined using aligned EBL and another round of Ar^+^-based reactive ion etching. Finally, the devices are cleaned and clad with PECVD silica once again. Finally, 2 cm-long gold microwave electrodes are defined near the waveguides using aligned EBL and photolithography and metalized using electron-beam deposition followed by a liftoff process. Facets at the edge coupler tips are exposed through reactive ion etching through the X-TFLN, thermal oxide, and silicon, resulting in a total EO phase modulator insertion loss of ~8 dB. The 6 GHz half-wave-voltage ${V}_{\pi }$ is about 4 V when optically pumped in the C-band. The nominal waveguide width is 1.5 μm, which minimizes radiative scattering losses while simultaneously preventing excess loss into higher-order waveguide modes as light propagates through the X-TFLN chip. Other relevant parameters are listed in the Supplementary Information.

### Microresonator dispersion engineering

The microresonator dispersion is defined by the integrated dispersion ${D}_{\mathrm{int}}\left(\omega \right)=\sum _{n\ge 2}\frac{{D}_{n}}{n!}{\left(\omega -{\omega }_{p}\right)}^{n}$. In the strong anomalous dispersion regime, ${D}_{2}\, >\, 0$ and ${D}_{2}\gg {D}_{n}$ for $n\ge 3$. The resulting DKS states emerging from microresonators in this regime have prototypical spectra featuring sech^2^ envelopes for the single DKS (Fig. 2a) and constructively/destructively interfering sech^2^ envelopes for multiple DKS. In the weak anomalous dispersion regime, ${D}_{2} \,>\, 0$ but ${D}_{n}$ for $n\ge 3$ are not small enough to neglect. This regime features dispersive-wave enhanced DKS states, characterized by energy transfer between the pump and phase-matched frequencies in the normal dispersion regime. Such behavior, in the temporal domain, is analogous to a Cherenkov-like emission of radiation by the DKS structure^72^. These states are critical in broadening DKS frequency combs beyond the anomalous dispersion regime and enabling an octave span (Fig. 2c). The dispersion parameters ${D}_{n}$ are directly obtained from eigenmode simulations of the fundamental transverse-electric-like mode’s (TE mode) effective refractive index ${n}_{{eff}}$, due to engineered microresonator waveguide cross-sections. We carry out such simulations using a commercial eigenmode solver (Lumerical MODE), including waveguide bending. The frequency-dependent ${n}_{{eff}}$ gives access to ${D}_{\mathrm{int}}$ and ${D}_{n}$ for all orders *n* through derivatives of ${n}_{{eff}}$ with respect to frequency. To design near-octave single DKS sources for the hybrid Kerr-EO architecture, we chose three ring radii (30, 40, and 50 μm), yielding about half-THz spacings. For each radius, we optimized the simulated ${D}_{\mathrm{int}}$ down to one representative microresonator waveguide width (1.48, 1.60, and 1.63 μm, respectively), while fixing the waveguide height to ~345 nm (Supplementary information Fig. S1a). For comparison, we also plot the ${D}_{\mathrm{int}}$ of the octave-spanning single DKS (Fig. 2c) in the same figure. The quasi-fundamental TE mode profiles are overlaid on top of each waveguide cross section. From fabricated microresonators with these designs, we measured DKS spectra using moderate on-chip powers between 100 and 250 mW (Supplementary information Fig. S1b-d, top row). Such spectra are broadened by dual dispersive waves at locations ${\omega }_{{DW}}$ where ${D}_{\mathrm{int}}\left({\omega }_{{DW}}\right) \sim 0$, consistent with simulations. As we decrease microresonator waveguide widths (1.48 to 1.44 *μ*m, 1.60 to 1.585 *μ*m, and 1.63 to 1.62 *μ*m, respectively), the total comb spans broaden as the dispersive wave locations move away from the pump, also consistent with expectation (Supplementary information Fig. S1b-d, bottom row). The good agreement between simulated and experimental dispersive wave positions suggests precisely controllable dispersion engineering for broadband DKS frequency combs on the Z-cut TFLN platform. We note that the microresonators in Supplementary information Fig. S1c-d were simultaneously engineered to overcome stimulated Raman scattering on TFLN, a rich topic of its own, which we point the reader to works that extensively explore this subject^73,74^.

### Microresonator dispersion measurement

In Fig. 2a, the experimental ${D}_{\mathrm{int}}$ in a narrow frequency band around the pump frequency is measured by fitting resonance frequencies of the microresonator and removing the fitted linear ${D}_{1}$. The resonance frequencies are calibrated by a fiber-based Mach-Zehnder interferometer with a fringe period of 191.3 MHz.

### DKS device characterization

The experimental setup for DKS device characterization and DKS generation is schematically illustrated in Supplementary Information Fig. S3. A continuous-wave (CW) pump laser is amplified by an erbium-doped fiber amplifier (EDFA). In Fig. 2a, a tunable bandpass filter was used to filter out the amplified spontaneous emission (ASE) associated noise with the pump power amplification. Further, a fiber-Bragg-grating (FBG) notch filter filters out the strong pump frequency component in the DKS spectrum. In Supplementary information Fig. S1b-d, the ASE was not removed from the spectrum and corresponds to the irregular shape beneath the DKS envelope. The pump was also not removed due to over ten meters of fiber in the notch filter, introducing excess fiber-induced losses below 150 THz. In both cases, polarization controllers and lensed fibers are used to couple light into the DKS chip. Lensed fibers are used to couple light from the DKS chip. One percent of the total output is used to monitor the fiber-to-chip in and out coupling. The remaining ninety-nine percent is then split such that ten percent is sent to a 125 MHz photodetector to monitor the total comb power as a voltage readout on an oscilloscope and ninety percent is sent to two optical spectrum analyzers (OSAs, covering 600–1700 nm and 1200–2400 nm) to simultaneously monitor the comb spectrum. Comb power measurements for DKS screening are taken by scanning the pump frequency back and forth through a microresonator resonance (Supplementary information Fig. 2) and are recorded as an oscilloscope voltage, noting that typically all available DKS states are accessible in the blue-to-red scan, yet only a subset of them are, in the red-to-blue scan. Here, the discrete step at about 0.77 (5.4 V) is only accessible sweeping from blue to red and corresponds to the single DKS in Fig. 2a. The stability measurement (Fig. 2b) consists of oscilloscope voltage values collected every 20 seconds over a total of 12 hours as the DKS state free-runs, starting from about 0.77 (5.4 V) at hour zero. In terms of DKS device lifetime, despite their uncladded designs, we did not notice meaningful changes in resonance quality factor up to time-scales of two months. However, during early stage DKS development, we have also observed device storage in environments without humidity control to reduce resonance quality factors over time-scales of weeks, though they can be completely restored following Piranha cleaning and annealing as described earlier.

### EO comb characterization

The experimental setup for integrated EO phase modulator performance characterization is schematically illustrated in Supplementary information Fig. S3. A CW pump laser passes through a polarization controller and is coupled on and off the EO phase modulator chip by lensed fibers. One percent of the total output is used to monitor the fiber-to-chip in and out coupling and polarization. The remaining ninety-nine percent is sent to an OSA (covering 600-1700 nm) to monitor the EO comb spectrum, resulting in those displayed in Fig. 3a, where five CW optical frequencies are sequentially coupled. The electrical power to generate these spectra is estimated to be about 4.47 W (36.5 dBm). This electrical power was chosen to demonstrate not only the efficiency but also the power-handling ability of our modulator chip, and a lower power level was used for hybrid Kerr-EO comb generation. Since the EO phase modulator is required to operate at all frequencies covering the source DKS span, our integrated phase modulator offers the uniquely existing on-chip solution while also being highly compact and power stable. We characterized its 6 GHz half-wave voltage (${V}_{\pi }$) and EO bandwidth at fifteen optical wavelengths in the telecommunications L, C, S, and E bands, only limited by available lasers, using the methodology described below. At each wavelength, 137 modulation frequencies (6 GHz to 40 GHz in steps of 0.25 GHz) are applied at power levels of 5 and 10 dBm output directly from a calibrated microwave source. For each modulation frequency and source output power combination, the EO comb spectrum is collected and the power in each comb line is extracted. The power of the *n*^th^ EO sideband theoretically corresponds to the *n*^th^ order Bessel function ${J}_{n}(\beta )$ evaluated at a modulation depth given by $\beta =\pi V/{V}_{\pi }$. Since the power delivered onto the modulator is related to *V*, we fit the EO sideband powers and delivered microwave power (source output power corrected for microwave circuit losses) to obtain the ${V}_{\pi }$ at all modulation frequency and source output power combinations. A single-valued ${V}_{\pi }$ for a given modulation frequency was obtained by averaging the two ${V}_{\pi }{\rm{s}}$ extracted when the source output power was varied between 5 and 10 dBm. Averaging over more power levels was not necessary as they always yielded nearly identical ${V}_{\pi }$, which further justifies averaging as only a means of reducing statistical variation in ${V}_{\pi }$ measurements at the same modulation frequency. Specifically, we repeated this ${V}_{\pi }$ vs. modulation frequency measurement at fifteen optical wavelengths of 1355 nm and 1370-1630 nm in intervals of 20 nm, and three representative curves are shown in Fig. 3b (labeled as optical frequencies). The first value of each curve is taken to be the 6 GHz ${V}_{\pi }$ (plotted as data points in Fig. 3c), and the modulation frequency at which the ${V}_{\pi }$ rises to $\sqrt{2}$ of its 6 GHz value is taken to be the EO 3 dB bandwidth (plotted as data points in Fig. 3d). We note that the measured ${V}_{\pi }$ displays slight dependence on the operational wavelength, outside of the mode-crossing-induced increase in ${V}_{\pi }$ near 1400 nm (215–220 THz in Fig. 3c, d). Shorter wavelengths (higher frequencies) generally feature slightly lower ${V}_{\pi }$, predominantly due to the wavelength-squared dependence itself, despite a reduced microwave-optical field overlap. These counterbalancing effects still lead to a rather uniform ${V}_{\pi }$ and enable spacing densification over the 75.9 THz hybrid Kerr-EO bandwidth, as evidenced in Fig. 4b. It is also worth noting that in X-TFLN EO phase modulators, the effect of velocity matching between the microwave phase velocity (around 29.5 GHz) and the optical group velocities across soliton bandwidths (spanning over 100 THz) has negligible impact on the modulation efficiency variation (see Supplementary information Fig. S4).

### Hybrid Kerr-EO comb generation

The experimental setup for hybrid Kerr-EO comb generation is schematically illustrated in Supplementary information Fig. S5. It is a combination of the separate setups for DKS generation and EO phase modulator characterization, except that the two chips are operated simultaneously, with an FBG notch filter to filter out the strong pump frequency component and a polarization controller linking the chips. Two OSAs are used to simultaneously monitor the comb spectrum. The measurement did not necessitate the use of the FBG, and the polarization controller may be omitted in conceivable monolithic integration or low-loss coupling schemes between Z-TFLN and X-TFLN, due to the good spatial mode overlap between the fundamental TE modes in both cuts of TFLN material.

To generate the hybrid Kerr-EO combs as in Fig. 4a, the optical power on-chip was estimated to be 125 mW from an EDFA output power of 500 mW, accounting for 6 dB coupling loss induced by the input facet. Notably, with bilayer taper edge couplers such as those fabricated on the EO phase modulator chip, the coupling loss may be reduced to 1.7 dB per facet, and the off-chip pump power requirement lowered to 188 mW^75^. Advanced coupler designs may be exploited for 0.54 dB loss per facet, and the off-chip pump power requirement further lowered to 142 mW^76^. Currently, the loaded quality factor (${Q}_{L}$) for our pump resonance in Fig. 4a is about 1 million. Fabrication improvements ${Q}_{L}$ by a factor of 2 may reduce the on-chip pump power requirement by a factor of four since our DKSs are operated far from the pump-to-comb conversion saturation regime. This translates to an on and off-chip power requirement of 31 mW and 35.5 mW, in the best case, which approaches power levels of state-of-the-art integrated laser transmitters in TFLN^57^. Packaged distributed feedback (DFB) lasers may also be butt-coupled to the DKS chip facet in combination with edge couplers to yield estimated facet losses conservatively around 3 dB, which translates to an on and off-chip power requirement of 31 mW and 62 mW, a regime compatible with commercial DFB laser output powers. The electrical power required to electro-optically divide 410.319 GHz of DKS spacing into microwave-rate (29.308 GHz) separated lines was calibrated to be 2.51 W (34.5 dBm) after accounting for microwave losses in the system, at 29.158 GHz modulation frequency. We specifically selected a microwave power such that the highest optical power would appear at the $N/2=7$ sideband. This choice ensures that the comb lines separated by the difference frequency $\Delta f$ would generate a microwave beatnote with a high signal-to-noise ratio, and phase locking $\Delta f$ to the stable microwave oscillator would yield a high-quality phase lock in the locking demonstration. The EO phase modulator measured in the C-band has a ${V}_{\pi }\cdot L \sim 8.84$ V⋅cm at 6 GHz modulation frequency and a 3 dB EO bandwidth of 31.47 GHz. The electrical power consumption may be further lowered by 5.3 dB using state-of-the-art TFLN EO phase modulators with ${V}_{\pi }\cdot L \sim 4.8$V$\cdot {\rm{cm}}$ (extrapolated from quoted $2.4{\rm{V}}\cdot {\rm{cm}}$ for a dual-drive amplitude modulator and 110 GHz 3 dB EO bandwidths^53^). This projects an electrical power on-chip of 0.24 W (23.8 dBm), reaching sub-Watt levels. Dual-drive phase modulators in a loop-back architecture may be utilized^54^, further lowering the projected electrical power on-chip by a factor of two down to 0.12 W (20.8 dBm), though in this case, additional care must be taken in microwave-optical velocity matching over the broad optical bandwidth.

The hybrid Kerr-EO comb inherits the combined simplicity and robustness of its components, resulting in exceptional performance metrics. This is due to the photorefraction-induced manual initiation and operational stability of Z-TFLN DKS generators, as well as the similar stability of ultra-efficient X-TFLN EO modulators, attributed to their non-resonant nature. The optical linewidth of each comb line in the hybrid Kerr-EO comb straightforwardly extends from theoretically and experimentally well-characterized properties of DKS^77^ and EO combs^28^. Microwave noise multiplication of the latter does not play a dominating role here due to the use of a commercial microwave source and the maximum EO sideband number being $N/2=7$.

### DKS spacing detection, locking, and stabilization

When generating the hybrid Kerr-EO comb, a nonzero difference frequency $\Delta f$ is introduced when ${f}_{{DKS}}$ is not an integer multiple of ${f}_{{RF}}$. Assuming $\Delta f$ is the difference frequency generated by the $N/2$ and $-N/2$ EO sidebands of DKS comb lines $n$ and $n+1$ respectively, $\Delta f={(f}_{{ceo}}+\left(n+1\right)\cdot {f}_{{DKS}}-\frac{N}{2}\cdot {f}_{{RF}})-({f}_{{ceo}}+n\cdot {f}_{{DKS}}+\frac{N}{2}\cdot {f}_{{RF}})$, simplifying to $\Delta f={f}_{{DKS}}-N\cdot {f}_{{RF}}$, which is the spacing relation given in the main text. Here, ${f}_{{ceo}}$ is the carrier-envelope offset frequency of the DKS comb. Further, the noise property of $\Delta f$ is given by $\delta \left(\Delta f\right)=\delta \left({f}_{{DKS}}\right)-N\cdot \delta ({f}_{{RF}})$. Since ${f}_{{RF}}$ is synthesized by a commercial microwave source, it fluctuates much less than the free-running ${f}_{{DKS}}$. Thus, we assume $\delta \left({f}_{{RF}}\right) \sim 0$ and the noise in $\Delta f$ reflects the noise in ${f}_{{DKS}}$ directly.

In our experiment in Fig. 4d (locking setup schematically illustrated in Supplementary information Fig. S5), $\Delta f$ was locked at 2.1078 GHz after detection by a 12 GHz bandwidth photodetector when ${f}_{{RF}}$ was set to 29.158 GHz. We note that this $\Delta f$ does not vary across the spectral span of the hybrid comb, since ${f}_{{DKS}}$ and ${f}_{{RF}}$ are fixed across its entire spectrum. The lock on $\Delta f$ was maintained by electronic feedback onto the current control of the pump laser, controlling its frequency while residual laser intensity fluctuations are negligible. During this lock, ${f}_{{DKS}}$ was fixed at 410.3198 GHz. To generate the $\Delta f$ beatnote on the photodetector, relevant EO sideband pairs were filtered and then amplified by a C-band pre-amplifier before beating on the photodetector. The $\Delta f$ was further amplified by a low-noise microwave amplifier (providing about 26 dB of gain) to produce a sufficient beatnote power level for the phase locked loop (PLL). The PLL is based on a phase comparator circuit where the reference frequency is 32 times a microwave oscillator set at 65.865 MHz. This oscillator shares the 10 kHz clock signal synthesizing ${f}_{{RF}}$. With a locked, nonzero $\Delta f$, each comb line is given by ${f}_{n,m}={f}_{{ceo}}+n\cdot {f}_{{DKS}}+m\cdot {f}_{{RF}}$, which is a three-variable comb structure. In particular, the frequency of each of the 2589 comb lines can be precisely known, once both ${f}_{{ceo}}$ and ${f}_{{DKS}}$ of the frequency combs are detected and stabilized. However, if ${f}_{{ceo}}$ cannot be extracted, then each comb line may be represented by ${f}_{{n}^{{\prime} },{m}^{{\prime} }}={f}_{{pump}}+{n}^{{\prime} }\cdot {f}_{{DKS}}+{m}^{{\prime} }\cdot {f}_{{RF}}$ instead, and the optical pump can be referenced for absolute frequency determination of each comb line. While the $\Delta f$-locked-state is not a two-variable comb in the conventional sense, access to known optical frequencies across a large bandwidth has broad usability in optical frequency metrology, multi-laser synchronization, among many other scenarios. Here, we have already demonstrated the possibility for the integrated detection, stabilization, and control of a near THz-rate ${f}_{{DKS}}$ using a single integrated EO phase modulator, which is naturally facilitated by the hybrid Kerr-EO approach.

### Comparison with other integrated frequency comb technologies

Comparing our work with state-of-the-art integrated frequency comb technologies across various material platforms, such as octave-spanning DKS combs and microresonator-based EO-combs, we find that the hybrid Kerr-EO comb of this work produces the largest span (75.9 THz) while simultaneously being capable of directly interfacing with conventional fast electronics, which we define as a sub-50 GHz spacing between adjacent comb lines. These features are augmented by a large number of 2589 comb lines produced using a comb generator consisting of purely integrated components. This comparison is graphically illustrated in Supplementary information Fig. S6. All data points are directly quoted from references or estimated from figures when exact numbers are not reported^24–28,31,35,36,38,78–88^. We note that substantial achievements were made in integrated frequency combs beyond DKS or EO combs as conventionally described, such as ones utilizing advanced dispersion engineering^89–91^ and additional nonlinear optical effects^92–94^, soliton crystals^95^, and lower operational power and turnkey microcombs^96–99^, which cannot be simply viewed by span, spacing, or number of comb lines.

## Supplementary information

Supplementary material for Hybrid Kerr-electro-optic frequency combs on thin-film lithium niobate

## Data Availability

All data needed to evaluate the conclusions in the paper are present the paper and/or the Supplementary Information. Supplementary information accompanies the manuscript on the Light: Science & Applications website.
