# Supplementary material for: Hybrid Kerr-electro-optic frequency combs on thin-film lithium niobate
Source: Light Sci Appl. 2025 Aug 12;14:270. doi: 10.1038/s41377-025-01906-x (PMC12339749; doi:10.1038/s41377-025-01906-x)
Supplement: Supplementary file 1 — Supplementary material for Hybrid Kerr-electro-optic frequency combs on thin-film lithium niobate [file 41377_2025_1906_MOESM1_ESM.pdf]

Supplementary information for: Hybrid Kerr-electro-optic frequency combs on thin-film lithium niobate

Yunxiang Song[1,2,*], Yaowen Hu[1], Marko Lončar[1,*], Kiyoul Yang[1,*]

[1]*John A. Paulson School of Engineering and Applied Sciences, Harvard University, Cambridge, MA 02138, USA*

[2]*Quantum Science and Engineering, Harvard University, Cambridge, MA 02138, USA*

*ysong1@g.harvard.edu, loncar@g.harvard.edu, kiyoul@seas.harvard.edu

This document contains Supplementary figures S1-S6.

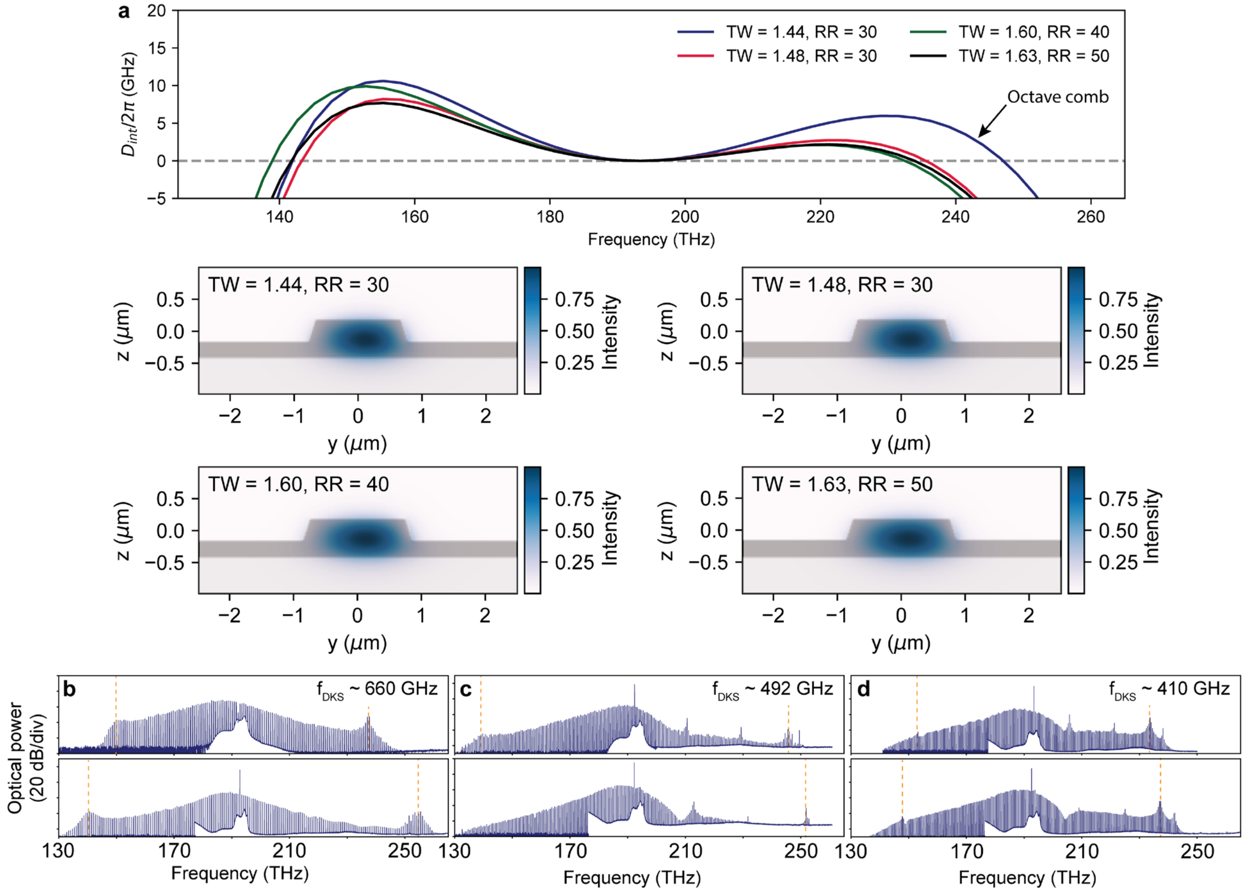

Fig. S1 | Dispersion engineering of dissipative Kerr solitons on thin-film lithium niobate. a, Simulated integrated dispersion $D_{int}/2\pi$ of four representative microresonators, yielding the octave-spanning spectrum in Fig. 2c, and near-octave-spanning spectra from 30, 40, and 50 μm-radius microresonators in panels **b-d** of this figure, respectively. In the latter cases, the waveguide parameters are tuned such that $D_{int}/2\pi$ are similar and correspond to sufficiently broadband DKS states. The different colors of $D_{int}/2\pi$ curves correspond to DKS spectra as follows: Fig. 2c and Fig. S1b bottom (blue); Fig. S1b top (red); Fig. S1c top (green); and Fig. S1d top (black). For each $D_{int}/2\pi$, the simulated E_y of the quasi-fundamental TE mode electric field distribution at about 193 THz is shown, illustrating the effects of waveguide bending and modal confinement on the field shape, hence on the geometric dispersion and effective index. **b-d**, Dispersion engineered 30, 40, and 50 μm-radius microresonators and single DKS spectra from them, corresponding to f_{DKS} of about 660, 492, and 410 GHz, respectively. The microresonator waveguide width is decreased from top to bottom rows and the dispersive wave locations (red dashed lines) move away from the pump frequencies, accordingly, indicating increased anomalous dispersion at the pump frequencies.

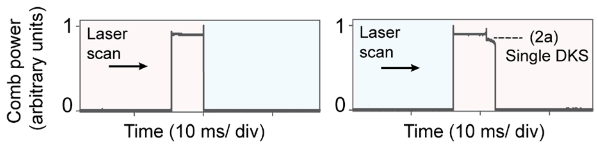

Fig. S2 | Dissipative Kerr soliton comb power. Total comb power traces as the pump laser scans across a DKS-hosting microresonator resonance, in the red to blue-detuned (left) and blue to red-detuned (right) directions. The discrete step around 0.77 (normalized arbitrary units) in the latter direction is the single DKS state in Fig. 2a. The vertical axis is normalized against the photoreceiver output voltage range, where 0.77 corresponds to about 5.4 V.

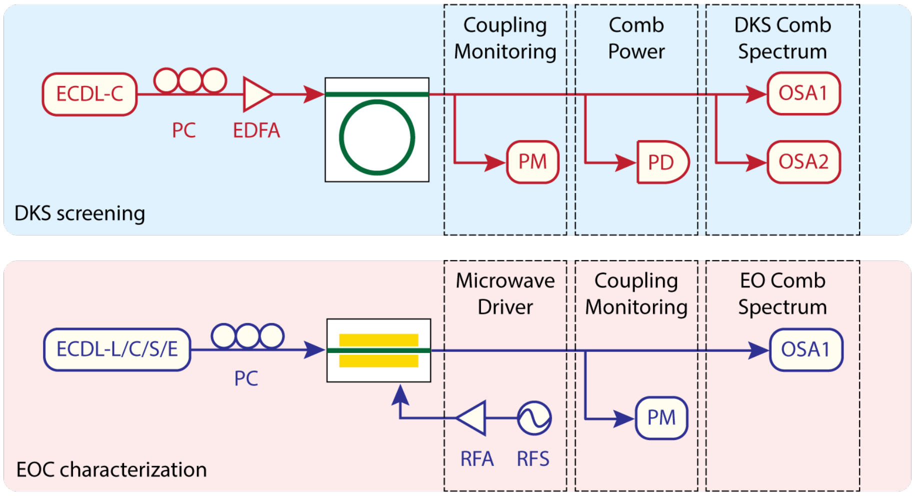

Fig. S3 | Dissipative Kerr soliton screening and electro-optic comb characterization setups. Blue (red) panel is the setup schematic for DKS screening (EO comb characterization). Abbreviations in the setup schematics are defined as follows. ECDL-L/C/S/E: external cavity diode laser covering the L/C/S/E-band. PC: polarization control paddles. EDFA: erbium-doped fiber amplifier. PM: optical power monitoring unit. PD: photodetector. OSA1/2: optical spectrum analyzers with 1200-2400 nm and 600-1700 nm spectral coverage, respectively. RFS: radiofrequency (microwave) source. RFA: radiofrequency (microwave) amplifier.

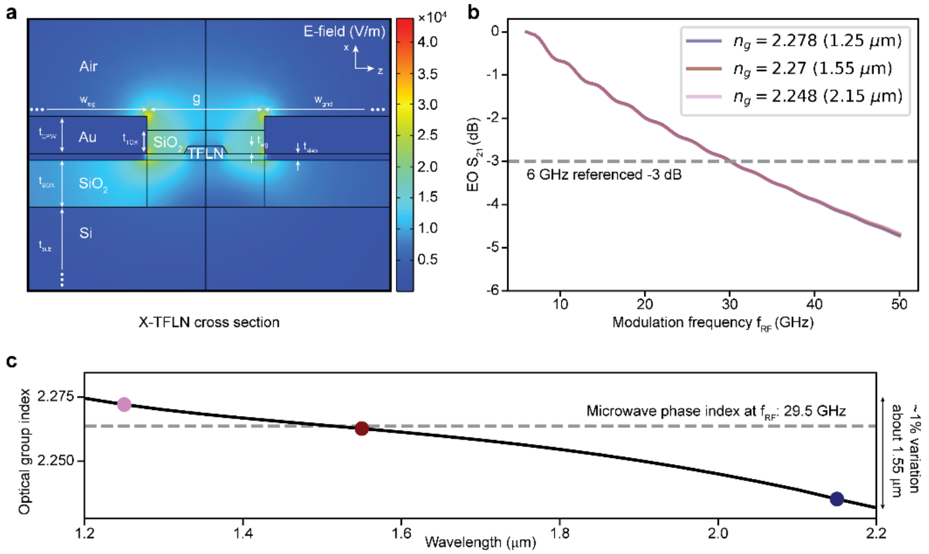

Fig. S4 | Integrated electro-optic comb design parameters and velocity matching. a, X-TFLN EO comb material cross section, and coplanar waveguide microwave mode at 29.5 GHz (normalized electric field, COMSOL). Material and parameter abbreviations, as well as corresponding numeric values are defined as follows. Au: gold. SiO_2: silica. Si: silicon. TFLN: thin-film lithium niobate. t_{CPW}: coplanar waveguide thickness, 1.6 μm. w_{sig}, w_{gnd}: coplanar waveguide signal and ground widths, 30 μm and 75 μm, respectively. g: coplanar waveguide signal to ground gap, 5 μm. t_{TOX}, t_{BOX}: top and bottom silica cladding thicknesses, 1.0 μm and 2.0 μm, respectively. t_{wg}, t_{slab}: X-TFLN waveguide and slab thicknesses, 320 nm and 280 nm, respectively. t_{sub}: silicon substrate thickness, 0.525 mm. The waveguide width and sidewall angle are 1.5 μm and 60 degrees, respectively. Minor oscillations are due to overall characteristic impedance mismatch with 50 Ω. **b**, Simulated modulation efficiency given by the EO S_{21} parameter, accounting for optical group velocity variation across the soliton bandwidth. As shown, the extent of this variation has negligible impact on the single-tone microwave modulation efficiency at 29.5 GHz. The zero-dB level is referenced to 6 GHz for consistency with Fig. 3b of the main text. **c**, Optical group index and microwave phase index matching across the soliton bandwidth. Sampled points at 1.25, 1.55, and 2.15 μm of wavelength correspond to the three modulation efficiency curves in **b**. Note that the variation in optical group index is just over 1 % about its corresponding value at 1.55 μm.

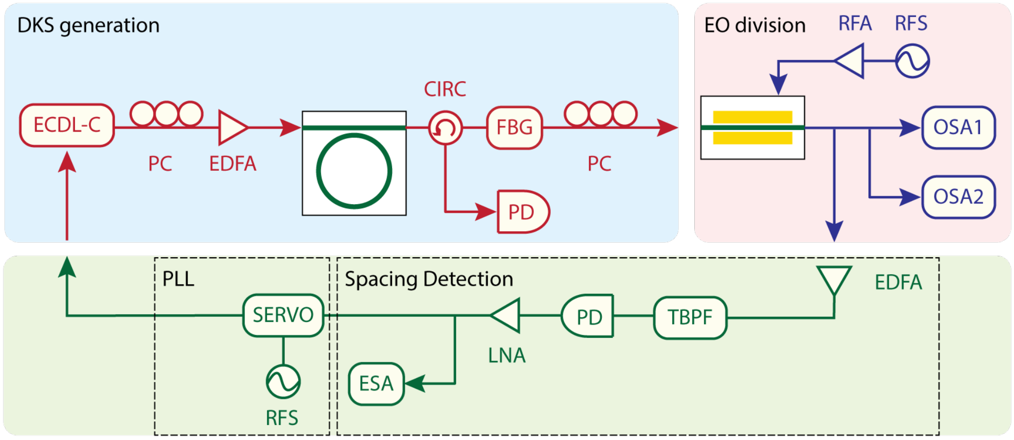

Fig. S5 | Hybrid Kerr-electro-optic comb generation and stabilization setup. Blue panel generates the DKS comb. The DKS comb is fed into the red panel for EO division, which divides the near THz-rate DKS spacing down to microwave rates. The hybrid Kerr-EO comb is then fed into the green panel, which (1) detects the difference frequency Δf between the $\pm 7^{\text{th}}$ order sidebands generated around adjacent DKS comb lines, and (2) phase locks Δf to a stable microwave oscillator. The phase lock is maintained by a comparative error signal that is fed back to the pump current for pump frequency adjustment. Abbreviations in the setup schematic are defined as follows. ECDL-C: external cavity diode laser covering the C-band. PC: polarization control paddles. EDFA: erbium-doped fiber amplifier. CIRC: circulator. FBG: fiber Bragg grating. PD: photodetector. OSA1/2: optical spectrum analyzers with 1200-2400 nm and 600-1700 nm spectral coverage, respectively. RFS: radiofrequency (microwave) source. RFA: radiofrequency (microwave) amplifier. TBPF: tunable optical bandpass filter. LNA: low-noise radiofrequency (microwave) amplifier. ESA: electrical spectrum analyzer. SERVO: electronic servo control. PLL: phase locked loop.

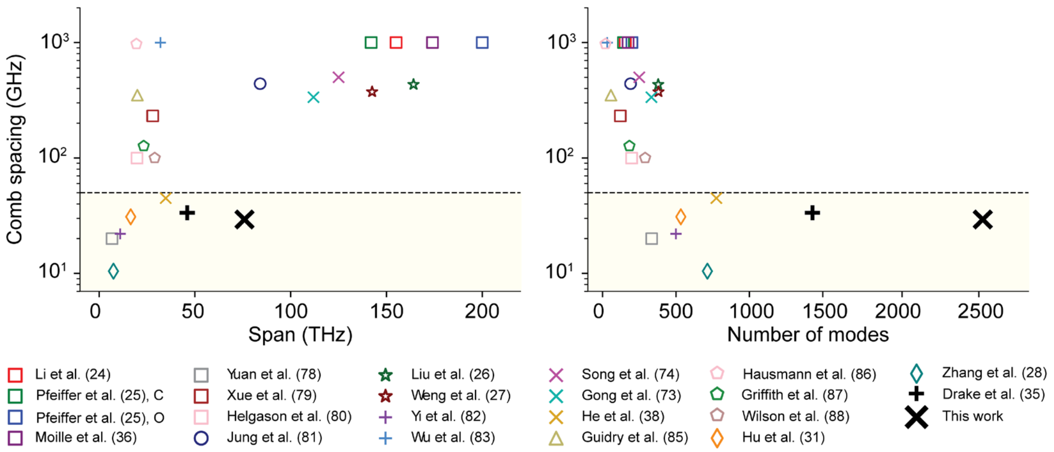

Fig. S6 | Comparison with other integrated optical frequency comb technologies. Our hybrid Kerr-EO frequency comb (black cross) compared to other integrated frequency comb generators, considering spacing, span, and number of modes. The black dashed line marks a spacing of 50 GHz and the shaded yellow region below represents the region that can be interfaced with conventional fast electronics.
